# Risk of new acute myocardial infarction hospitalization associated with use of oral and parenteral non-steroidal anti-inflammation drugs (NSAIDs): a case-crossover study of Taiwan's National Health Insurance claims database and review of current evidence

**DOI:** 10.1186/1471-2261-12-4

**Published:** 2012-02-02

**Authors:** Wen-Yi Shau, Hsi-Chieh Chen, Shu-Ting Chen, Hsu-Wen Chou, Chia-Hsuin Chang, Chuei-Wen Kuo, Mei-Shu Lai

**Affiliations:** 1 Division of Health Technology Assessment, Center for Drug Evaluation, 3F, No.465, Sec. 6, Zhongxiao E. Rd., Taipei 115, Taiwan; 2 Graduate Institute of Epidemiology and Preventive Medicine, College of Public Health, National Taiwan University, 5F18. No. 17, Hsuchow Road, Taipei 100, Taipei, Taiwan; 3 Department of Internal Medicine, National Taiwan University Hospital, No.7, Chung Shan S. Rd., Taipei 100, Taiwan; 4 Medical Review and Pharmaceutical Benefits Division, Bureau of National Health Insurance, Department of Health, Executive Yuan, 3F, No.3, Beipin W. Rd., Taipei 100, Taiwan; 5 Center for Comparative Effectiveness Research, National Clinical Trial and Research Center, National Taiwan University Hospital, 17 Hsuchow Road, Taipei 100, Taipei, Taiwan

## Abstract

**Background:**

Previous studies have documented the increased cardiovascular risk associated with the use of some nonsteroidal anti-inflammatory drugs (NSAIDs). Despite this, many old NSAIDs are still prescribed worldwide. Most of the studies to date have been focused on specific oral drugs or limited by the number of cases examined. We studied the risk of new acute myocardial infarction (AMI) hospitalization with current use of a variety of oral and parenteral NSAIDs in a nationwide population, and compared our results with existing evidence.

**Methods:**

We conducted a case-crossover study using the Taiwan's National Health Insurance claim database, identifying patients with new AMI hospitalized in 2006. The 1-30 days and 91-120 days prior to the admission were defined as case and matched control period for each patient, respectively. Uses of NSAIDs during the respective periods were compared using conditional logistic regression and adjusted for use of co-medications.

**Results:**

8354 new AMI hospitalization patients fulfilled the study criteria. 14 oral and 3 parenteral NSAIDs were selected based on drug utilization profile among 13.7 million NSAID users. The adjusted odds ratio, aOR (95% confidence interval), for risk of AMI and use of oral and parenteral non-selective NSAIDs were 1.42 (1.29, 1.56) and 3.35 (2.50, 4.47), respectively, and significantly greater for parenteral than oral drugs (p for interaction < 0.01). Ketorolac was associated with the highest AMI risk among both of oral and parenteral NSAIDs studied, the aORs were 2.02 (1.00, 4.09) and 4.27 (2.90, 6.29) respectively. Use of oral flurbiprofen, ibuprofen, sulindac, diclofenac, and parenteral ketoprofen were also significantly associated with increased AMI risk. The results of the present study were consistent with the majority of evidence from previous studies.

**Conclusions:**

The collective evidence revealed the tendency of increased AMI risk with current use of some NSAIDs. A higher AMI risk associated with use of parenteral NSAIDs was observed in the present study. Ketorolac had the highest associated risk in both oral and parenteral NSAIDs studied. Though further investigation to confirm the association is warranted, prescribing physicians and the general public should be cautious about the potential risk of AMI when using NSAIDs.

## Background

Non-steroidal anti-inflammatory drugs (NSAIDs) are commonly used medications for reducing inflammation and relief pain. Due to large population exposed to NSAIDs, the risk of serious adverse cardiovascular effect for patient taking NSAIDs is an area of concern, from both a clinical and public health perspective [1-9]. Despite the frequent prescription of a wide variety of old oral and parenteral NSAIDs, recent studies exploring links between cardiovascular risk and NSAIDs use, including randomized controlled trials and observational studies, had mostly focused on cyclooxygenase-2 selective inhibitors (COX-2) or some non-selective NSAIDs (ns-NSAIDs), and were limited by the number of cases examined [1-3,10-12]. The assessment of the risk of acute myocardial infarction (AMI) from the use of parenteral NSAIDs was not a focus of the studies. The aim of this study was to assess the risk of hospitalization due to AMI as a result of the use of a variety of oral and parenteral NSAIDs in outpatient-clinic settings, and to compare the results with existing evidence.

## Methods

### Data Source

We used the claims database of Taiwan's National Health Insurance (NHI) for the present study. The NHI is a universal compulsory program launched in March 1995 by the Taiwan government. More than 98% of the total 23 million populations was covered by NHI at the end of year 2005 [13]. Out-patient clinic and in-patient hospitalization services provided by both of private and public sectors were included in a unified reimbursement system. All medical claims were submitted and captured electronically. The complete history of diagnosis (using International Classification of Disease, 9^th ^Revision, Clinical Modification, ICD-9-CM code), prescriptions, procedure, and examination ordered for every patient could be identified and traced by civil identification number. To comply with the personal electronic data-privacy regulation, personal identities were encrypted and all data were analysed anonymously. The study protocol was approved by the Research Ethics Committee of National Taiwan University Hospital.

In order to compare the results of the present study with previous studies, we summarized previously published observational studies and reviews of randomized controlled trials (RCTs). The collection of observational studies was based on previous reviews [1,2,9,11,14-16] and extended to the first half of year 2010. We summarized a total of 33 observational studies (10 cohort [17-26], 21 case control [27-47] and 2 case-crossover [17,18]) and the results of five reviews of RCTs with meta-analysis or pooled subjects analysis [3,5,10,12,48] which had compared the AMI risk of current use of NSAIDs (celecoxib and ns-NSAIDs) to placebos or non-users. Studies that focused on COX-2 other than celecoxib, such as rofecoxib or lumiracoxib were not included because these NSAIDs were not marketed in Taiwan during the study period covered by the present study.

### Study design

We used a case-crossover design to study the association between current use of NSAIDs and AMI hospitalization risk. This design is similar to a matched case-control study [49-51]. One of the challenges of using a matched case-control study is the selection of control subjects. Potential risk factors of outcomes may be different between case subjects and control subjects. Some of these factors may not be assessed in the study, leading to less accurate study results. In the case-crossover design, we used past exposure experience as the patient's own matched control (the control period) and compared this with one's own exposure status immediately prior to onset of the AMI event (the case period). By using the same patient to make our comparisons, we were able to avoid the issue of between-subject risk-factor differences, both measured and unmeasured. This choice of study design is appropriate when the exposure is intermittent, the effect on outcome is immediate and transient, the outcome event is abrupt, and the patients remain relatively stable during study period. We found the NSAIDs were prescribed for intermittent and short term use in most of the patients of the present study (additional file 1, table S1). We test the effect of 30-day short-term current use of NSAIDs on new AMI hospitalization during a 120-day study period, within which period the health status of studied patients remained relatively stable. Thus, the case-crossover design is appropriate for the purpose of the present study.

### Population and study subjects

The target outcome of this study was new AMI hospitalization. We searched all the medical claims of NHI beneficiaries aged between 20 to 100 years old in order to identify the first hospitalized AMI (ICD-9-CM code 410) in 2006. To be eligible, patients needed to be continuously covered by NHI in 2005 and 2006. The date of the first AMI hospitalization was defined as the index date. Patients with any clinical visit, emergency-room visit or hospitalization for myocardial infarction (ICD-9-CM code 410 or 412) either 365 days preceding the index date or in 2005 were excluded. To increase the comparability of general health condition between the case and control period of a patient, those who had been hospitalized for any cause during 120 days prior to their index date were also excluded.

### Drug use and potential time-varying confounding factors

The primary exposure of the present study included the COX-2 selective inhibitor and non-selective NSAIDs, which were reimbursed by NHI in Taiwan in 2005 and 2006. All prescriptions were searched for oral and parenteral single-active-ingredient NSAIDs and were classified according to the Anatomic Therapeutic Chemical (ATC) classification system [52]. Drugs in the class M01A (anti-inflammatory and anti-rheumatic products, non-steroids) were included, excepting glucosamine (M01AX05). We included all drugs within the top 95 percentile used by patients in 2006. Prescription history was summarized as either exposed or not exposed during the paired case and control periods of each patient for every NSAID studied. Cumulative dosage of a drug prescribed within the case or control period was measured using defined daily dose (DDDs) [52], and divided by number of days of the period to calculate the mean DDDs per day to explore the possible dose response. Drugs were analysed individually, as well as classified by administration rout (oral and parenteral) and by COX-2 selectivity (selective and non-selective). We also collected data on age, gender, other medications prescribed concomitantly, and the diagnoses associated with the NSAIDs prescription. The concomitant medications were included as within-patient time-varying potentially confounding factors for adjusted analysis. Patients were further grouped by hypertension diagnosis and by use of low-dose aspirin during study period, in order to explore the potential modification effects of these factors.

### Statistical analysis

The association between risk of AMI hospitalization and use of NSAID was analysed by comparing the NSAID exposure status between the paired case and the control period of each patient. If a patient had not used an NSAID in the control period but had used it in the case period, there was a positive association between AMI hospitalization and current use of the NSAID. The association between AMI and NSAID would be negative if an NSAID was used in the control period but not in the case period. When a patient used an NSAID in both of case and control periods, or did not use an NSAID in either period, the association between AMI and the NSAID were neutral. Conditional logistic regression was used to analyse the paired data set with case and control period as dependent variables, and use or non-use of an NSAID as independent variables. Concomitant medications were considered as time-varying cofactors and were included to allow adjustment for potential within-patient time-varying confounding of effects. In addition to the primary analysis, we performed sensitive analyses by altering the case period to 8-30 days and the control period to 98-120 days prior to the index date; and by changing the control period to 61-90 days prior to the index date. We used the PHREG procedure, which is a feature of SAS statistics software (version 9.1, SAS Institute Inc., Cary, NC, USA), to calculate the crude and adjusted odds ratio (aOR) and their associated 95% confidence intervals (95%CI). A 2-sided p value of less than 0.05 was considered statistically significant.

## Results

**Figure 1:**
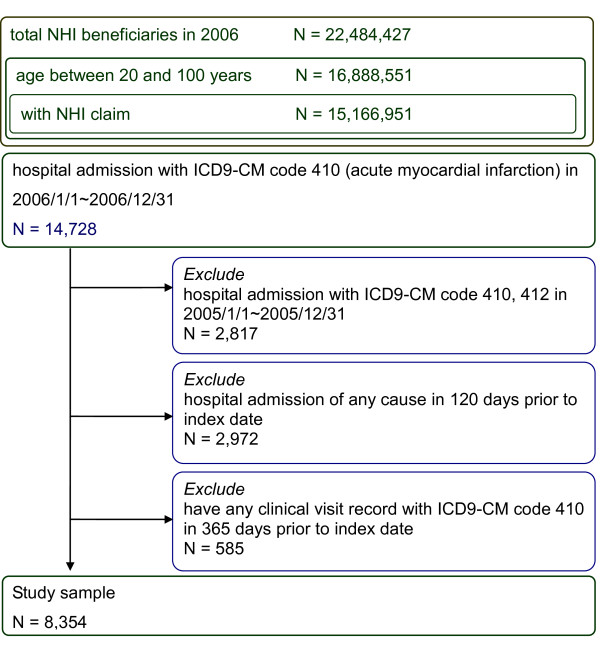
**Patients flow diagram**. NHI = National Health Insurance, ICD-9-CM = International Classification of Disease, 9thRevision, Clinical Modification.

Among the 22,484,427 NHI beneficiaries (98.3% of residential population of Taiwan), we identified 14,728 patients hospitalized with the principle diagnosis of AMI between 1^st ^January and 31^st ^December 2006. We excluded 2,817 patients with previous MI diagnosis in 2005; 2,972 patients had been hospitalized for any cause during the 120 days prior to their index date; and the other 585 patients had outpatient visits with AMI diagnosis 365 days preceding their index date. The final analysis included 8,354 patients with a new AMI hospitalization (Figure 1). Their mean age was 65.5 ± 13.8 (SD) years old, and 71.3% were male. Their comorbidities and concomitant medications during the case and control period were summarized in Table 1. Hypertension was the most frequent condition (42% in the case period and 37% in the control period) followed by diabetes mellitus (23% in the case period and 22% in the control period). There was a higher frequency of comorbidities and more concomitant medications prescribed in the case period than the control period.

**Table 1:** Comorbidities and concomitant medications during 1-30 days and 91-120 days prior to new AMI hospitalization

N = 8,354	Case period 1-30 days before index day	Control period 91-120 days before index day
Comorbidity (%)		
Hypertension	41.81	36.65
Diabetes mellitus	23.23	21.58
Osteoarthritis	8.55	7.79
Peptic ulcer disease	7.94	7.17
Chronic lung disease	6.48	6.12
Congestive heart failure	4.30	3.51
Chronic renal disease	3.17	2.82
Chronic liver disease	3.10	2.98
Atrial fibrillation	2.21	1.86
Rheumatoid arthritis	0.81	0.72
Migraine	0.65	0.41
Cancer	0.16	0.16
Concomitant medication (%)		
calcium channel blocker	27.16	24.34
ACE inhibitors or ARBs	23.93	20.68
beta-blocker	22.06	17.05
sulfonylurea	15.75	14.97
statins	11.31	10.16
low dose aspirin	7.23	7.88
antihypertensive agents	5.12	4.31
insulin	4.39	3.58
thiazolidinediones	2.54	2.56
glinides	1.83	1.62
loop diuretics	1.59	1.28
non-aspirin antiplatelet agents	1.11	1.21
vitamin k antagonist	0.81	0.67

ACE: angiotensin converting enzyme; ARB: angiotensin receptor blocker

The NHI drug-reimbursement list in 2006 shows 1009 oral and 119 parenteral single-active-ingredient NSAID items in ATC class M01A, "anti-inflammatory and anti-rheumatic products, non-steroids", excluding glucosamine (M01AX05). 13,699,038 patients received at least one NSAID prescription. Fourteen NSAIDs were selected for the present study according to the top 95 percentile user number. They were diclofenac (M01AB05, 29.3%), mefenamic acid (M01AG01, 21.8%), ibuprofen (M01AE01, 20.6%), naproxen (M01AE02, 4.4%), acemetacin (M01AB11, 3.8%), piroxicam (M01AC01, 2.7%), flurbirofen (M01AE09, 2.1%), sulindac (M01AB02, 2.0%), indomethacin (M01AB01, 1.9%), meloxicam (M01AC06, 1.6%), tiaprofenic acid (M01AE11, 1.5%), ketorolac (M01AB05, 1.3%), ketoprofen (M01AE03, 1.1%), and celecoxib (M01AH01, 1.0%). Celecoxib was the only COX-2 selective NSAID reimbursed during 2005 and 2006 in Taiwan. Though the number of patients using celecoxib was not large, the quantity used in terms of number of DDDs accounted for 6.15% of total NSAIDs DDDs used in 2006.

Table 2 shows the association between current use of NSAID and new AMI hospitalization. The crude ORs (95%CIs) for celecoxib, oral and parenteral ns-NSAID overall were 1.47 (1.05, 2.07), 1.53 (1.40, 1.68), 5.08 (3.89, 6.62) respectively. When adjusted for use of other NSAIDs and concomitant medications as potential time-varying confounders, the aOR for celecoxib and AMI decreased slightly to a non-significant 1.36 (0.95, 1.96). The aORs for oral and parenteral ns-NSAIDs overall remained statistically significant. These were 1.42 (1.29, 1.56) and 3.35 (2.50, 4.47) respectively. The aOR for parenteral NSAIDs and AMI was much higher than that of oral NSAIDs, the two 95%CIs were not overlapped, and the difference in aORs was significant (p < 0.01). Higher risks in parenteral NSAIDs were observed for all the three drugs with both of oral and parenteral form - ketorolac, ibuprofen, and diclofenac. Except for parenteral diclofenac, owing to limited exposed patient numbers, the increased risks of parenteral NSAIDs were statistically significant.

**Table 2:** Risk of new AMI hospitalization associated with current use of COX-2 selective and non-selective NSAIDs

	**Number of patients exposed to the drug during**			**Crude**		**Adjusted**			
	**study period**	**case period but not control period**	**control period but not case period**	OR*	**95%CI**	**OR^+^**	**95%CI**	**OR^$^**	**95%CI**
		
**Oral**									
celecoxib	210	81	55	1.47	1.05 - 2.07	1.42	0.99 - 2.04	1.36	0.95 - 1.96
non-selective NSAIDs									
ketorolac	76	43	11	3.91	2.02 - 7.58			2.02	1.00 - 4.09
flurbiprofen	129	54	29	1.86	1.19 - 2.92			1.71	1.06 - 2.74
ibuprofen	755	278	178	1.56	1.29 - 1.89			1.45	1.19 - 1.76
sulindac	242	93	61	1.53	1.10 - 2.11			1.44	1.02 - 2.03
diclofenac	1,731	624	421	1.48	1.31 - 1.68			1.29	1.13 - 1.47
acemetacin	349	136	93	1.46	1.12 - 1.90			1.28	0.96 - 1.70
naproxen	231	78	56	1.39	0.99 - 1.96			1.26	0.88 - 1.81
piroxicam	259	94	76	1.24	0.91 - 1.67			1.25	0.92 - 1.70
indomethacin	184	67	44	1.52	1.04 - 2.23			1.22	0.82 - 1.83
ketoprofen	70	28	22	1.27	0.73 - 2.23			1.17	0.64 - 2.11
mefenamic acid	1,070	370	294	1.23	1.08 - 1.47			1.16	0.98 - 1.36
meloxicam	327	106	96	1.10	0.84 - 1.46			0.97	0.73 - 1.30
tiaprofenic acid	92	31	28	1.11	0.66 - 1.85			0.91	0.52 - 1.57
						
ns-NSAIDs overall	3,573	1,175	768	1.53	1.40 - 1.68	1.42	1.29 - 1.56		
**Parenteral**									
ketorolac	364	245	35	6.99	4.91 - 9.96			4.27	2.90 - 6.29
ketoprofen	125	69	20	3.45	2.10 - 5.68			2.34	1.31 - 4.19
diclofenac	70	34	12	2.83	1.47 - 5.46			1.88	0.95 - 3.75
						
Parenteral NSAIDs overall	455	330	65	5.08	3.89 - 6.62	3.35	2.50 - 4.47		

OR = odds ratio; 95%CI = 95% confidence interval; ns-NSAIDs = non-selective NSAIDs

* Crude analysis for oral drug users excluded those ever used parenteral drugs during study period; same exclusion of oral drugs use for parenteral users.

+ Conditional logistic regression for all patients of celecoxib, oral ns-NSAIDs overall, and parenteral ns-NSAIDs overall and adjusted for potential time-varying confounding variables of all discordant use of antihypertensive agents, angiotensin converting enzyme inhibitors or aldosterone receptor blockers, calcium channel blockers, statins, insulin, sulfonylurea, thiazolidinedione, and aspirin between case and control period.

$ Conditional logistic regression for all 17 drugs included in one model with adjustment of above mentioned time-varying confounding variables.

Among the individual NSAIDs, ketorolac posed the highest risk for AMI in both of oral and parenteral NSAIDs. The aORs (95%CI) were 2.02 (1.00, 4.09) and 4.27 (2.90, 6.29) respectively. In addition to oral and parenteral ketorolac, the aORs for oral flurbiprofen, ibuprofen, sulindac, diclofenac, and parenteral ketoprofen were also statistically significantly greater than 1, ranging from 1.29 to 1.71 for oral drugs, and 2.34 for parenteral ketoprofen. The aORs for the rest of the other eight oral ns-NSAIDs and parenteral diclofenac were not statistically significant. No significantly protective effect to prevent new AMI hospitalization was observed for any NSAID studied.

Table 3 shows the results from the subgroup of patients with or without a hypertension diagnosis, and use or non-use of low-dose aspirin during the study period. The aORs for AMI remained significant for both oral and parenteral ns-NSAIDs overall in all patient subgroups and the higher AMI risk of parenteral NSAIDs compared with oral NSAIDs were consistently observed within all subgroups.

**Table 3:** Adjusted odds ratio and 95% confidence interval for new AMI hospitalization with NSAIDs stratified by hypertension and use of low dose aspirin

	**Hypertension diagnosis**					**Low dose aspirin**				
		
	**with**		**without**		**p value** **for interaction**	**user**		**non-user**		**p value for interaction**
	**(N = 3,672)**		**(N = 4,682)**			**(N = 905)**		**(N = 7,449)**		
	aOR*	**95%CI**	aOR*	**95%CI**		aOR*	**95%CI**	aOR*	**95%CI**	
			
**ns-NSAIDs**										
Oral overall	1.56	1.36 - 1.79	1.32	1.15 - 1.51	0.05	1.48	1.10 - 1.99	1.42	1.28 - 1.57	0.93
Parenteral overall	3.43	2.30 - 5.13	3.18	2.08 - 4.87	0.95	4.95	1.96 - 12.50	3.24	2.38 - 4.40	0.93
**Oral**										
celecoxib	1.81	1.07 - 3.05	1.10	0.66 - 1.83	0.51	1.66	0.67 - 4.10	1.36	0.91 - 2.02	0.43
diclofenac	1.33	1.11 - 1.60	1.26	1.04 - 1.52	0.50	1.17	0.77 - 1.76	1.30	1.13 - 1.49	0.68
naproxen	1.30	0.81 - 2.10	1.17	0.67 - 2.04	0.83	2.66	0.89 - 7.91	1.15	0.78 - 1.69	0.27
ketorolac	7.64	1.74 - 33.47	0.86	0.36 - 2.09	0.03	1.30	0.21 - 8.02	2.19	1.01 - 4.75	0.57
**Parenteral**										
ketorolac	4.96	2.82 - 8.71	3.71	2.16 - 6.39	0.62	7.47	2.31 - 24.21	4.06	2.69 - 6.13	0.66
ketoprofen	2.73	1.18 - 6.31	1.92	0.83 - 4.46	0.36	1.20	0.09 - 16.21	2.38	1.31 - 4.35	0.39
diclofenac	1.08	0.44 - 2.65	3.84	1.09 - 13.52	0.15	2.28	0.22 - 24.06	1.86	0.90 - 3.83	0.91

aOR = adjusted odds ratio; 95%CI = 95% confidence interval; ns-NSAIDs = non-selective NSAIDs

* Conditional logistic regression adjusted for important potential time-varying confounding variables of all discordant use of antihypertensive agents, angiotensin converting enzyme inhibitors or aldosterone receptor blockers, calcium channel blockers, statins, insulin, sulfonylurea, thiazolidinedione, and aspirin between case and control period

For individual NSAID in the subgroups, the aOR (95%CI) of celecoxib was significant only in patients with hypertension, which was 1.81 (1.07, 3.05). The aORs of diclofenac were all significant, except in the low-dose-aspirin user subgroup. No aORs of individual oral NSAIDs were significant in the low-dose-aspirin user subgroup; but parenteral ketorolac had significant aOR of 7.47 (2.31, 24.21) for AMI in this subgroup. Most of the effects of NSAIDs on AMI were not substantially modified by hypertension diagnosis or use of low-dose-aspirin. Their 95%CIs in separate patient groups overlapped and the p values for testing interaction were greater than 0.15. The exceptions were observed for oral ns-NSAIDs overall and oral ketorolac in particular. Their effects on risk of AMI were higher in hypertensive patients than in patients without hypertension, and the p values test for interaction were 0.05 and 0.03 respectively. We did not find significantly modified effects by gender or age.

Table 4 shows the result of association between AMI and use of high dose (DDD per day ≥ 0.5) or low dose (DDD per day between 0 and 0.5) NSAIDs. The adjusted ORs of the use of high dose were all statistically significant, and the strength of association was consistently stronger than that of the low dose used in the same drug category revealed the trend of dose response.

**Table 4:** Association of new AMI hospitalization and current use of NSAIDs by mean dose of NSAIDs used per day

NSAIDs used (DDDs per day)	Crude OR	95% CI		Adjusted OR*	95% CI	
Oral celecoxib						
low dose (> 0, < 0.5)	1.11	0.15 -	7.91	1.39	0.18 -	10.70
high dose (≥ 0.5)	1.49	1.05 -	2.11	1.47	1.02 -	2.12
Oral ns-NSAIDs overall						
low dose (> 0, < 0.5)	1.22	0.85 -	1.76	1.12	0.76 -	1.65
high dose (≥ 0.5)	1.65	1.42 -	1.93	1.56	1.32 -	1.83
Parenteral ns-NSAIDs overall						
low dose (> 0, < 0.5)	3.77	2.73 -	5.19	2.96	2.12 -	4.14
high dose (≥ 0.5)	14.60	3.45 -	61.87	11.79	2.73 -	50.99

95%CI = 95% confidence interval; ns-NSAIDs = non-selective NSAIDs; DDD = defined daily dose

* Conditional logistic regression adjusted for important potential time-varying confounding variables of all discordant use of antihypertensive agents, angiotensin converting enzyme inhibitors or aldosterone receptor blockers, calcium channel blockers, statins, insulin, sulfonylurea, thiazolidinedione, and aspirin between case and control period

The sensitivity analyses generated by altering case and control periods did not change the significant of the aORs of oral and parenteral ns-NSAIDs overall, and the higher risk with parenteral NSAIDs remained (table 5). The associations for celecoxib and AMI remained non-significant. The aORs of oral and parenteral ketorolac remained significant in all the analyses. Strength of association with AMI and associated statistical significance were altered for parenteral diclofenac and ketoprofen, and for oral diclofenac in the analyses by removing the proximate seven days prior to index date, or by using 61 to 90 days prior to index date as the control period.

**Table 5:** Sensitivity analysis of adjusted odds ratio and 95% confidence interval for new AMI hospitalization and NSAIDs use on different definition of case and control period

	**days before index date**					
**Case period** **Control period**	**1-30** **91-120**		**8-30** **98-120**		**1-30** **61-90**	
	aOR*	**95%CI**	aOR*	**95%CI**	aOR*	**95%CI**
		
**ns-NSAIDs**						
Oral overall	1.42	1.29 - 1.56	1.19	1.08 - 1.31	1.48	1.35 - 1.63
Parental overall	3.35	2.50 - 4.47	1.85	1.34 - 2.57	2.81	2.13 - 3.70
**Oral**						
celecoxib	1.42	0.99 - 2.04	1.19	0.83 - 1.71	1.03	0.73 - 1.46
ketorolac	2.02	1.00 - 4.09	2.58	1.25 - 5.33	2.37	1.23 - 4.59
diclofenac	1.29	1.13 - 1.47	1.13	0.99 - 1.30	1.46	1.28 - 1.66
**Parenteral**						
ketorolac	4.27	2.90 - 6.29	1.98	1.25 - 3.13	2.90	2.05 - 4.10
ketoprofen	2.34	1.31 - 4.19	1.77	0.97 - 3.22	1.98	1.12 - 3.50
diclofenac	1.88	0.95 - 3.75	1.14	0.53 - 2.47	2.35	1.09 - 5.08

aOR = adjusted odds ratio; 95%CI = 95% confidence interval; ns-NSAIDs = non-selective NSAIDs

* Conditional logistic regression adjusted for time-varying confounding variables of discordant use of antihypertensive agents, angiotensin converting enzyme inhibitors or aldosterone receptor blockers, calcium channel blockers, statins, insulin, sulfonylurea, thiazolidinedione, and aspirin between case and control period.

Table 6 and 7 summarizes the evidence of association between AMI risk and current use of NSAIDs from results of previous studies and the present study.

**Table 6:** Evidence of association between MI risk and current use of NSAIDs

**Reference**	**Outcome**	**ns-NSAIDs**			**Celecoxib**			**Naproxen**			**Diclofenac**			**Ibuprofen**			**Indomethacin**			**Meloxica**		
**Observational studies**		n*	**aOR**	**95%CI**	n*	**aOR**	**95%CI**	n*	**aOR**	**95%CI**	n*	**aOR**	**95%CI**	n*	**aOR**	**95%CI**	n*	**aOR**	**95%CI**	n*	**aOR**	**95%CI**
						
**Case-Crossover**																						
Present study	AMI	3573	1.42	1.3, 1.6	210	1.36	1.0, 2.0	231	1.26	0.9, 1.8	1731	1.29	1.1, 1.5	755	1.45	1.2, 1.8	184	1.22	0.8, 1.8	327	0.97	0.7, 1.3
Gislason 2009 [17]	MI in HF	-	1.56	1.2, 2.1	-	1.31	0.9, 2.0	-	1.31	0.6, 2.7	-	1.64	1.2, 2.3	-	1.47	1.2, 1.9	-			-		
Gislason 2006 [18]	re MI	14	1.07	0.8, 1.5	42	1.36	0.7, 2.5	-			61	1.67	1.2, 2.4	136	1.32	0.7, 2.5	-			-		
**Case-Control**																						
Garcia Rodriguez 2000 [44]	MI	167	1.45	1.2, 1.8	-			-			-			-			-			-		
Schlienger 2002 [43]	AMI	242	1.17	1.0, 1.4	-			19	0.68	0.4, 1.1	97	1.38	1.1, 1.8	60	1.17	0.9, 1.6	15	1.03	0.6, 1.9	-		
Solomon 2002 [42]	AMI	390	0.86	0.6, 1.2	-			243	0.84	0.7, 1.0	-			285	1.02	0.9, 1.2	-			-		
Watson 2002 [41]	TCEs	-	1.16	0.9, 1.5	-			26	0.61	0.4, 0.9	-	1.68	1.3, 2.3	-	1.05	0.6, 1.7	-			-		
	MI	-	1.47	1.0, 2.2	-			-	0.57	0.3, 1.1	-	1.68	1.1, 2.5	-	0.74	0.4, 1.6	-			-		
Garcia Rodriguez 2004 [40]	AMI	580	1.07	1.0, 1.2	-			49	0.89	0.6, 1.2	213	1.18	1.0, 1.4	155	1.06	0.9, 1.3	29	0.86	0.6, 1.3	25	0.97	0.6, 1.6
Kimmel 2004 [27]	AMI (no aspirin)	126	0.53	0.4, 0.7	-			-	0.48	0.3, 0.8	-			-	0.52	0.4, 0.7	-			-		
	(use aspirin)	74	0.83	0.6, 1.2	-			-			-			-			-			-		
Solomon 2004 [39]	AMI	371	0.98	-	425	0.93	0.8, 1.0	63	0.98	-	-			49	0.95	-	-			-		
Fischer 2005 [28]	AMI	650	1.07	1.0, 1.2	-			63	0.96	0.7, 1.4	260	1.23	1.0, 1.5	176	1.16	0.9, 1.5	36	1.36	0.8, 2.3	-		
Graham 2005 [38]	AMI, CD	534	1.13	1.0, 1.3	126	0.84	0.7, 1.0	367	1·14	1.0, 1.3	-			670	1·06	1.0, 1.2	-			-		
Hippisley-Cox 2005 [37]	AMI	181	1.21	1.0, 1.4	93	1.21	1.0, 1.5	96	1.27	1.0, 1.6	542	1.55	1.4, 1.7	460	1.24	1.1, 1.4	-			-		
Johnsen 2005 [36]	AMI	532	1.68	1.5, 1.9	71	1.25	1.0, 1.6	26	1.50	1.0, 2.3	-			-			-			-		
Kimmel 2005 [29]	MI	319	0.61	0.5, 0.7	18	0.43	0.2, 0.8	-			-			-			-			-		
Levesque 2005 [35]	AMI	51	1.00	0.7, 1.4	287	0.99	0.9, 1.2	23	1.17	0.8, 1.8	-			-			-			7	1.06	0.5, 2.3
Andersohn 2006 [34]	AMI	354	1.14	1.0, 1.2	111	1.56	1.2, 2.0	59	1.15	0.8, 1.6	393	1.37	1.2, 1.6	201	1.04	0.9, 1.3	-			-		
Hawkey 2006 [33]	MI (cc)	36	1.77	1.0, 3.0	-			-			-			-			-			-		
	(hc)	36	2.61	1.4, 5.0	-			-			-			-			-			-		
Helin-Salmivaara 2006 [32]	MI	1985	1.34	1.3, 1.4	124	1.06	0.8, 1.3	300	1.19	1.0, 1.4	388	1.35	1.2, 1.5	768	1.41	1.3, 1.6	108	1.56	1.2, 2.0	149	1.06	1.0, 1.6
Jick 2006 [45]	AMI	-			62	1.40	0.9, 2.1	44	1.40	0.8, 2.3	94	1.00	0.8, 1.4	102	1.2	0.9, 1.6	-			-		
Brophy 2007 [46]	AMI	51	1.01	0.7, 1.4	287	1.03	0.9, 1.2	23	1.18	0.8, 1.8	-			-			-			7	0.88	0.4, 1.9
Garcia Rodriguez 2008 [31]	MI	940	1.34	1.2,1.5	81	1.33	1.0, 1.8	54	1.04	0.7, 1. 5	353	1.67	1.4, 1.9	143	1.06	0.9, 1.3	29	1.47	0.9, 2.4	59	1.30	0.9, 1.8
van der Linden 2009 [47]	AMI	269	1.41	1.2, 1.6	18	2.53	1.5, 4.2	-	1.21	0.9, 1.7	108	1.51	1.2, 1.9	68	1.56	1.2, 2.1	-			-		
Mangoni 2010 [30]	AMI^+^	1803	1.02	1.0, 1.1	-			284	0.93~1.31^+^	608	0.82~1.04^+^	274	0.92~1.32^+^	-			679	0.81~1.21^+^				
**Cohort**																						
Ray 2002 [26]	AMI, CD	-			74	0.96	0.8, 1.2	245	0.93	0.8, 1.1	-			190	0.91	0.8, 1.1	-			-		
Ray 2002 [25]	AMI, CD	841	1.05	1.0, 1.1	-			201	0.95	0.8, 1.1	-			339	1.15	1.0, 1.3	-			-		
Mamdani 2003 [24]	AMI	134	1.20	0.9, 1.4	75	0.90	0.7, 1.2	15	1.00	0.6, 1.7	-			-			-			-		
Chan 2006 [23]	CV	288	1.44	1.3, 1.7	-			-			-			-			-			-		
Gislason 2006 [18]	re MI	14	1.27	1.1, 1.5	42	1.50	1.1, 2.1	-			61	1.54	1.2, 1.9	136	1.25	1.1, 1.5	-			-		
Solomon 2006 [22]	AMI, stroke	292	0.95	0.8, 1.1	1342	0.99	0.9, 1.1	108	0.75	0.6, 0.9	86	1.1	0.9, 1.4	151	0.96	0.8, 1.1	-			-		
Hammad 2008 [21]	AMI	18	1.33	0.8, 2.2	-			-			-			-			-			-		
van Staa 2008 [20]	AMI	5690	1.12	1.1, 1.2	-			526	1.03	0.9, 1.1	2033	1.21	1.2, 1.3	1913	1.04	1.0, 1.1	309	1.27	1.1, 1.4	137	1.12	0.9, 1.3
Roumie 2009 [19]	CV (no CV history)	-			300	1.00	0.9, 1.1	424	1.00	0.9, 1.1	57	1.02	0.8, 1.3	355	1.03	0.9, 1.2	95	1.36	1.1, 1.7	-		
	CV (CV history)	-			312	0.92	0.8, 1.0	306	0.88	0.8, 1.0	49	1.01	0.8, 1.3	281	1.02	0.9, 1.2	60	0.97	0.8, 1.3	-		
Gislason 2009 [17]	MI in HF	-	1.32	1.1, 1.5	-	1.38	1.1, 1.7	-	1.52	1.1, 2.1	-	1.36	1.1, 1.6	-	1.33	1.2, 1.5	-			-		

**Table 7:** Evidence of association between MI risk and current use of NSAIDs

	**n trials**	**outcome / **	**total**			**Celecoxib**			**Naproxen**			**Diclofenac**			**Ibuprofen**		
**Review of RCTs**		**(population)**	n*	**ratio^$^**	**95%CI**	n*	**ratio^$^**	**95%CI**	n*	**ratio^$^**	**95%CI**	n*	**ratio^$^**	**95%CI**	n*	**ratio^$^**	**95%CI**
					
Kearney 2006[3]	121 RCTs	vascular events							-	0.92	0.7, 1.3	-	1.63	1.1, 2.4	-	1.51	1.0, 2.3
	combined COX2s	MI	113	1.86	1.3, 2.6												
	41 celecoxib trials	MI				44	>1.8	p < 0.05									
																	
Salpeter 2006[12]	13 RCTs	CV events	33	1.3	0.8, 2.1												
	6 naproxen trials	(joint disease)							2	0.7	0.2, 2.5						
	2 naproxen trials	(Alzheimer)							26	1.5	0.9, 3.0						
																	
White 2007[10]	39 RCTs	nonfatal MI															
		(adjudicated)				5	1.56	0.2, 11.9									
		(non-adjudicated)				7	1.24	0.3, 5.8									
																	
Chen 2008[5]	40 celecoxib trials	MI and CV events															
	5 placebo control	(at least 1 event)					1.10	0.4, 3.5									
	4 placebo control	(200mg/day)					0.85	0.2, 3.2									
	3 placebo control	(400mg/day)					2.98	0.6, 14.7									
	2 placebo control	(800mg/day)					1.04	0.2, 7.5									
																	
Solomon 2008[48]	6 RCTs	Composite CV event															
		(overall adjusted)				101	1.7	1.1, 2.3									
		(400 mg QD)				30	1.1	0.6, 2.0									
		(200 mg Bid)				38	1.8	1.1, 3.1									
		(400 mg Bid)				33	3.1	1.5, 6.1									

aOR = adjusted odds ratio; aRR = adjusted rate ratio

TCEs = thromboembolic cardiovascular events; CD = cardiac/coronary heart disease death; AMI = acute myocardial infarction; MI = myocardial infarction; re MI = recurrent MI; HF = heart failure; CV = cardiovascular events

(cc) = (community control); (hc) = (hospital control); RCTs = randomized controlled trials

* n: number of exposed subjects with outcome

- not reported

+ range of adjusted odds ratios in 4 drug supplies categories: 1~4, 5~10, 11~19, > 20 NSAID supplies in last 2 years

$ ratio could be either one of RR(rate ratio), OR (odds ratio), or HR(hazard ratio)

## Discussion

We found that the risk of new AMI hospitalization was increased with the current use of oral ns-NSAIDs overall. The risk was higher with parenteral ns-NSAIDs overall. The associations between AMI risk and individual oral ns-NSAID were mild to moderate, and not significantly increased for celecoxib with adjusted analysis. We did not find any specific NSAID with significantly protective effect to prevent new AMI hospitalization.

### Parenteral NSAIDs and ketorolac

The new AMI hospitalization risk in users of parenteral ns-NSAIDs overall was significantly higher than that observed in oral ns-NSAIDs users. This higher risk of parenteral ns-NSAIDs remained for subgroups categorized by status of hypertension diagnosis and use of low-dose-aspirin, and was consistent across sensitivity analyses.

We found the use of ketorolac, in both oral and parenteral forms, was associated with the highest risk for new AMI hospitalization among all the NSAIDs. In 2006, 766,948 patients (about 3.3% of total population) received parenteral ketorolac, and more than one million DDDs (1 DDD = 30 mg) of injectable ketorolac were reimbursed by NHI (additional file 1 table S4). It was the most popular parenteral NSAID prescribed. Arora et al. [53] point out that although "physicians [might hold the] belief that parenteral administration of ketorolac are more effective than oral administration of ibuprofen", their review shows this belief to be false. Kimmel et al. [54] reported a reduced MI risk for parenteral ketorolac in comparison with use of parenteral opioids in a matched hospitalized cohort. Their study reported 18 MI events out of 10,219 courses of ketorolac treatment and 45 MI events out of 10,145 opioid treatment courses, by using propensity score adjusted analysis. Their inpatient setting was different from that of the present study, however. And it would be difficult to discern from the study whether this result was due to a lower risk of MI in the ketorolac group, or a higher risk in opioids group if compared to no use of either one. The subjects in our study received their NSAIDs in outpatient clinics, and we compared risks of AMI associated with use and non-use of various NSAIDs. These reasons may explain the different in study results.

Given the off-patent status of ketorolac and many generic products are currently available, thus it would be difficult to initiate well-controlled randomized trials with sufficient subject number to examine the safety of parenteral ketorolac in a outpatient clinic population. Consequently, large scale observational studies may be the only feasible information source. In our study, there were 364 new AMI patients exposed to parenteral ketorolac before their hospitalization. This may be the largest exposed AMI-patient series of this kind so far.

There were other safety concerns related to ketorolac [55], such as acute renal failure [56] and stroke [57]. Due to reports of anaphylactic shock leaded to patient death with use of ketoroloac, the Department of Health of Taiwan government has ruled that a new warning must be added to the package insert of all ketorolac-containing products from 2008 onwards. Because of these safety concern, and because other NSAIDs are available, in Taiwan parenteral ketorolac is now used only with patients for whom oral intake is contraindicated, and should be used for no more than five days [Department of Health, regulatory document (in Chinese)] [58]. Nevertheless, AMI risk related to the use of ketorolac in a general population has not been mentioned until now.

### Celecoxib

Whit [10] conducted a meta-analysis of 39 RCTs of celecoxib which were compared to a placebo or other ns-NSAIDs for cardiovascular risk. They found no significant difference in non-fatal AMI risk for celecoxib compared to other ns-NSAIDs. Our study reports similar findings. We found that the strength of adjusted association between current use of celecoxib and the risk of new AMI hospitalization was a moderate aOR of 1.36. It was comparable to the aORs with other oral ns-NSAIDs in the present study, and also within the range of non-fatal AMI relative risk of 1.24 to 1.56 for celecoxib compared to placebo in White's analysis. However, the total number of patients in the celecoxib group with MI was limited to 79 for all the 39 trials included in the meta-analysis [10]. This limitation may have affected the results. In our study, by using the 210 identified celecoxib users with new AMI hospitalization, the crude OR was 1.47 and statistically significant, but this reduced to a non-significant 1.36 with adjusted analysis. Similar significant crude and non-significant adjusted associations were seen in the other large scale population based observational studies [24,32,36-38,59], as well as non-significant results [19,22,26,30,35,38,39]. On the other hand, significantly increased AMI risk with current use of celecoxib were also observed in studies from the United Kingdom [34], the Netherlands [47], and in patients with previous MI from Canada [46]. A further study reported significantly reduced AMI risk [44]. The meta-analysis of 13 observational studies by McGettigan reported neutral cardiovascular risk with celecoxib [11]. Significantly increased risk for composite cardiovascular events with celecoxib compared to a placebo has been reported in the "Adenoma Prevention with Celecoxib" trial, which found 18 patients with non-fatal MI incidence in the celecoxib group [60]. The meta-analysis of 121 RCTs by Kearney reported a significantly increased summary risk of MI for five COX-2 selective inhibitors as a group compared to the placebo (rate ratio = 1.86, p < 0.01). The analysis including 41 trials for celecoxib, and no significant heterogeneity (p = 0.9) between the effects of individual drugs studied was found [3]. The pooled analysis of individual subjects from six RCTs by Solomon found increased risk for composite cardiovascular risk with celecoxib [61]. Chen looked at five celecoxib versus placebo trials for MI events, and found no significant association. Though in a summary of another nine trials, they report that the risk was higher for celecoxib compared to other NSAIDs [5]. The controversy surrounding the relationship between use of celecoxib and cardiovascular risk seems to be based on evidence cumulated to date. The result of the large scale trial, "Prospective Randomized Evaluation of Celecoxib Integrated Safety vs Ibuprofen or Naproxen" (PRECISION, ClinicalTrials.gov Identifier: NCT00346216), expected in 2013, may shed further light on this issue.

### Oral ns-NSAIDs

Several previous studies reported moderately increased AMI risk with statistical significant for current (< 30 days) or recent (< 3 months) use of ns-NSAIDs or NSAIDs overall [20,23,31-33,36-38,43,44,47]. Some other study results did not show statistical significant [22,24,25,30,34,35,40,42,43,46]. Aside from two case-control studies based on telephone interviews with patients concerning their use of NSAIDs [27,29], no other studies have reported significantly protective effects against AMI with ns-NSAIDs use in general. Though the difference in patient characteristics, clinical settings, study design, types and use of NSAIDs studied might have impact on the association between ns-NSAIDs use and AMI risk, current evidence collectively shows a tendency towards increased AMI risk for current or recent ns-NSAIDs users by studies using either cohort or case-control design. By using a case-crossover design in present study we found a 42% relative increase of AMI risk, which was consistent with the range of most adjusted relative associations, from 5% reduction to 77% increase of risk in previous studies. The four studies reported different results were as follows: two found a significant 39% and 47% relative reduction of risk based on telephone interviews [29]; one reported a non-significant 14% relative decrease of risk [42]; and one reported a significant 161% relative increase of risk by using a hospital control group [33].

Among the individual ns-NSAIDs, for current us of naproxen: three previous studies reported significant reduction of AMI risk [19,22,41]; two studies reported significantly increased AMI risk [37,38]; and a number of studies showed no statistically significant associations [19,20,24-26,31,34-36,39-41,43,46,47]. Salpeter conducted a meta-analysis of 13 RCTs of ns-NSAIDs, 8 of which were for naproxen, and found no significant effect on cardiovascular events or death with a pooled OR (95%CI) of 1.3 (0.8, 2.1) [12]. In the present study, the aOR (95%CI) of naproxen was 1.26 (0.88, 1.81) - a similar level of association as the meta-analysis result. The effect of naproxen on the risk of AMI could be considered as neutral-to-marginal increase.

Except for naproxen, no significant reduction of AMI risk has been reported for current or recent use of other individual ns-NSAID in previous studies. This was also the case in our study. We did not observe a significant protective effect of any individual NSAID in preventing AMI hospitalization in any subgroup of patients. Nevertheless, significantly increased AMI risk with use of diclofenac has been repeatedly reported in previous studies [18,20,28,31,34,37,41,43,47] and most of the non-significant results showed a trend of elevated AMI risk with diclofenac [19,22,62]. A meta-analysis of 10 observational studies for diclofenac on cardiovascular risk conducted by McGettigan, showed an increased pooled summary relative risk of 1.40 (1.19, 1.65) [11]. The aOR (95%CI) of diclofenac in our study was a significant 1.29 (1.13, 1.47). Though the strength of association was not as strong as the meta-analysis result, it was in the same direct and with a similar level of association. Diclofenac could therefore be said to slightly increase the risk of AMI. Diclofenac was the most widely prescribed NSAIDs in Taiwan. Of more than 7 million patients, account for one third of total population had received at least one prescription in 2006 (additional file 1 table S4). Our results suggest that use of diclofenac should be managed with caution from public health point of view.

Consistent with four previous studies which found a significantly increased AMI risk with current use of ibuprofen [18,25,37,47], we found a significant aOR of 1.45 for ibuprofen in the present study. The level of association was within the range of 9% reduction to 56% elevation reported in previous studies [18-20,22,25,26,31,34,37,38,42,43,47,62]. Ibuprofen is frequently prescribed in Taiwan and other countries [63-65]. Attention should be paid to the relative safety and benefit of such extensive use.

Flurbiprofen was another oral ns-NSAID associated with significantly elevated AMI risk in the present study. Few studies had previously evaluated this drug specifically. One study reported a non-significant 2.26 times increase of first-time AMI risk with flurbiprofen in a population based case-control study, but with only 22 users of this drug [43].

One more oral ns-NSAID significantly associated with AMI risk in the present study was sulindac, with aOR (95%CI) of 1.44 (1.02, 2.03). In the case-control study by Mangoni, users of sulindac and oxicams were pooled together and no association with AMI was found [30]. However, the effects of sulindac, piroxicam, and meloxicam in the present study were not very close. The risks of AMI associated with piroxicam or meloxicam in the present study were not significant. If all three drugs had been considered as one group, the results would have been non-significant. Previous studies also reported non-significant associations for piroxicam, and meloxicam [20,31,35,40,43,46]. More information is required to elucidate the cardiovascular risk of above four drugs.

### Interactions between use of NSAIDs and use of low dose aspirin or hypertension diagnosis

We did not find significant interaction effects between the use of NSAIDs and low-dose aspirin on AMI risk. Both significant and non-significant interaction results had been reported in previous studies [5,27,31,48]. Limited number of patients used low-dose aspirin, and the possible over-the-counter use of low-dose aspirin could lead to statistical imprecision and reduce our ability to test for the interactions in the present study. More evidence is required in order to evaluate the effect of NSAIDs on cardiovascular risk in patients taking low-dose aspirin regularly.

The interaction between NSAIDs use and hypertension in patients was also a concern [66]. A fairly high AMI risk with oral ketorolac was observed in patients with hypertension diagnosis, the aOR (95%CI) was 7.64 (1.74, 33.47), and the test for interaction of NSAID by hypertension was significant (p = 0.03). The AMI risk with oral ns-NSAIDs overall was also higher in patients with hypertension than those without hypertension in the present study. Though not all the results were statistically significant, the effects on risk of AMI tended to be higher in hypertensive patients than those without hypertension for most of the individual ns-NSAIDs in the present study. Consider hypertension a strong indicator of cardiovascular risk, patients with higher cardiovascular risk may be more susceptible to the AMI risk with use of ns-NSAIDs. This trend consistent with previous case-control study by Brophy [46] and the pooled subjects analysis of six RCTs by Solomon [61]. It seems that the higher cardiovascular risk effect of NSAIDs for patients with higher background cardiovascular risk may not be limited to clelcoxib. Caution should be taken when prescribing NSAIDs to patients with higher background cardiovascular risk.

### Study population and study design

The database used in this study covered the entire population of Taiwan. This not only provided large patient pool but also enabled follow up of each patient, thus minimizing the problem of case ascertainment. Owing to the large population pool, subjects recruited within a year were sufficient for most of the drugs studied, and more individual drugs could be analysed in one study. By limiting the recruitment period to one year, we were able to limit the potential influence of environmental changes, such as introduction of new drugs, change in medical practice, and epidemic trends of AMI in the community. The NHI reimburses a comprehensive list of prescription drugs, thus reducing the possibility and proportion of patients taking over-the-counter NSAIDs.

An important issue for most non-randomized studies using cohort or nested case-control design to analyse AMI risk with use of NSAIDs is the challenge of selection of appropriate comparison subjects. The unmeasured risk factors of outcomes may differ between subjects in the comparison groups, leading to a confounded result. The case-crossover design of the present study utilized a within-subject comparison, removing the potentially confounding by between-subjects difference [49], has also been applied to study the risk of NSAIDs in other studies [17,18,67]. Some risk factors, like body weight, smoking status, alcohol consumption, family history of cardiac vascular disease, were not captured in the database. These factors do not change substantially in the short term for a single patient, however, and thus a within-subject comparison balances their impact on the outcome. Though the case-crossover design is an efficient design, it may be sensitive to the time widow selected for the analysis. The sensitivity analyses of the present study revealed robust results for the main findings. The 30-day before new AMI hospitalization period selected in this study was similar to that used in previous cohort or case-control studies for current or recent use of NSAIDs [17,18,21,22,30-32,46]. Though the outcome of AMI events used in this study was based on the claimed diagnosis, medical charts were regularly audited by clinical experts when claims were submitted as part of routine NHI reimbursement process. Hospitalization was used to increase the specificity of outcome measurement. This outcome definition might lead to the inclusion of sever subjects only and underestimate the effect. By using the prescription records of a claim database, we were unable to measure the level of compliance of patients taking the medication prescribed, which could lead to non-differential misclassification and may bias association toward null. This should not be a concern for parenteral use of NSAIDs, however, as patients received their injections in clinics under prescribing doctor's direct supervision. As a result, the difference between effects of oral and parenteral NSAIDs seen in the present study may be exaggerated.

## Conclusion

The collective evidence revealed the tendency of increased AMI risk with current use of some NSAIDs. A higher AMI risk associated with use of parenteral NSAIDs was observed in the present study. Ketorolac had the highest associated risk in both oral and parenteral NSAIDs studied. Though further investigation to confirm the association is warranted, prescribing physicians and the general public should be cautious about the potential risk of AMI when using NSAIDs.

## Competing interests

The authors declare that they have no competing interests.

## Authors' contributions

All authors read and approved the final manuscript. The contributions of each author are:

WYS conceived and designed the study, analysed and interpreted the data and drafted the manuscript; HCC had full access to all the data, took responsibility for the integrity of the data, carried out the statistical analysis and provided administrative support; STC had full access to all the data, took responsibility for the integrity of the data, carried out the statistical analysis and provided administrative support; CHC conceived and designed the study, analysed and interpreted the data, critically review and revised the manuscript; HWC conceived and designed the study, analysed and interpreted the data, provided administrative support, critically review and revised the manuscript; CWK conceived and designed the study, analysed and interpreted the data, critically review and revised the manuscript; MSL obtained funding, conceived and designed the study, analysed and interpreted the data, had full access to all the data, took responsibility for the integrity of the data, critically review and revised the manuscript and supervised the study.

## Pre-publication history

The pre-publication history for this paper can be accessed here:

http://www.biomedcentral.com/1471-2261/12/4/prepub

## Supplementary Material

Additional file 1 **supplementary tables**. There are 4 additional tables (table S1 to table S4) in the file to present the utilization pattern of the NSAIDs studied. Table S5 is the STROBE checklist for the present study. Click here for file
