# Supplementary material for: Risk of new acute myocardial infarction hospitalization associated with use of oral and parenteral non-steroidal anti-inflammation drugs (NSAIDs): a case-crossover study of Taiwan's National Health Insurance claims database and review of current evidence
Source: BMC Cardiovasc Disord. 2012 Feb 2;12:4. doi: 10.1186/1471-2261-12-4 (PMC3395814; doi:10.1186/1471-2261-12-4)
Supplement: Additional file 1 — supplementary tables. There are 4 additional tables (table S1 to table S4) in the file to present the utilization pattern of the NSAIDs studied. Table S5 is the STROBE checklist for the present study. [file 1471-2261-12-4-S1.DOC]

Table S1: NSAIDs utilization 120 days before index acute myocardial infarction hospitalization among patients in Taiwan in 2006 (N=8,354)

| Drug | Route of administration | Number of items | Number of patients exposed (%) | Cumulative dosage (DDDs) (SD) | Cumulative days supplied (SD) | Mean DDDs per prescription (SD) | Mean DDDs per day (SD) | Number of patients with prescription < 2 (%) | |
| --- | --- | --- | --- | --- | --- | --- | --- | --- | --- |
| celecoxib | Oral | 2 | 210 (2.5) | 62.33 (68.02) | 57.45 (49.84) | 23.33 (13.93) | 1.05 (0.43) | 128 | (61) |
| diclofenac | Oral | 149 | 1,731 (20.7) | 13.89 (19.34) | 15.74 (22.65) | 5.11 (4.53) | 1.09 (0.48) | 1078 | (62) |
|  | Parenteral | 30 | 70 (0.8) | 0.97 (0.62) |  | 0.74 (0.31) | 0.30 (0.20) | 63 | (90) |
| mefenamic acid | Oral | 116 | 1,070 (12.8) | 8.05 (9.56) | 9.11 (15.16) | 3.60 (2.15) | 1.14 (0.50) | 728 | (68) |
| ibuprofen | Oral | 204 | 755 (9.0) | 7.07 (9.37) | 10.62 (17.51) | 3.00 (2.19) | 0.85 (0.32) | 505 | (67) |
| ketorolac | Oral | 5 | 76 (0.9) | 8.05 (14.64) | 10.50 (18.88) | 4.82 (5.55) | 0.95 (0.30) | 63 | (83) |
|  | Parenteral | 23 | 364 (4.4) | 1.28 (0.79) |  | 1.07 (0.38) | 0.45 (0.27) | 327 | (90) |
| meloxicam | Oral | 20 | 327 (3.9) | 27.65 (29.82) | 40.86 (41.03) | 10.68 (8.23) | 0.74 (0.31) | 194 | (59) |
| ketoprofen | Oral | 52 | 70 (0.8) | 18.95 (46.09) | 16.53 (31.44) | 8.19 (14.49) | 0.99 (0.44) | 47 | (67) |
|  | Parenteral | 32 | 125 (1.5) | 0.76 (0.92) |  | 0.44 (0.19) | 0.17 (0.14) | 102 | (82) |
| piroxicam | Oral | 79 | 259 (3.1) | 15.83 (19.01) | 10.76 (14.64) | 6.12 (3.83) | 1.64 (0.65) | 195 | (75) |
| sulindac | Oral | 44 | 242 (2.9) | 17.65 (23.37) | 26.58 (34.20) | 7.81 (7.22) | 0.82 (0.34) | 166 | (69) |
| naproxen | Oral | 75 | 231 (2.8) | 19.06 (29.39) | 15.81 (25.07) | 8.06 (8.93) | 1.46 (0.59) | 149 | (65) |
| acemetacin | Oral | 1 | 349 (4.2) | 13.40 (17.43) | 18.68 (25.17) | 6.38 (5.17) | 0.86 (0.28) | 271 | (78) |
| flurbiprofen | Oral | 7 | 129 (1.5) | 10.99 (16.59) | 16.45 (24.07) | 5.27 (5.53) | 0.86 (0.37) | 95 | (74) |
| Indomethacin | Oral | 64 | 184 (2.2) | 8.94 (15.76) | 18.32 (27.10) | 3.51 (4.78) | 0.67 (0.27) | 119 | (65) |
| tiaprofenic acid | Oral | 14 | 92 (1.1) | 14.42 (18.72) | 17.84 (23.54) | 7.45 (6.46) | 0.88 (0.25) | 71 | (77) |

DDD = Defined daily dose

SD = standard deviation

Table S2: Most frequent diagnosis and concomitant medication with NSAID prescription within 120 days before index hospitalization date among acute myocardial infarction patients in Taiwan in 2006

| Drug | Route of administration | Patients exposed | ICD9-CM diagnosis code with NSAID prescription (n) | | | | | |  | ATC code of co-medication together with NSAID prescription (n) | | | | | |
| --- | --- | --- | --- | --- | --- | --- | --- | --- | --- | --- | --- | --- | --- | --- | --- |
|  |  |  | 1st |  | 2nd |  | 3rd |  |  | 1st |  | 2nd |  | 3rd |  |
| celecoxib | Oral | 210 | 715.90 | (38) | 714.0 | (33) | 715.36 | (27) |  | A02AA02 | (54) | A07EC01 | (44) | M05BA04 | (43) |
| diclofenac | Oral | 1,731 | 465.9 | (365) | 724.2 | (279) | 724.5 | (202) |  | M03BB03 | (666) | N02BE01 | (556) | R05FA02 | (472) |
|  | Parenteral | 70 | 401.9 | (62) | 733.00 | (62) | 715.90 | (29) |  | N02BE01 | (17) | B05XA03 | (16) | M01AG01 | (12) |
| mefenamic acid | Oral | 1,070 | 465.9 | (249) | 784.0 | (99) | 463 | (71) |  | N02BE01 | (331) | R05FA02 | (286) | J01CA04 | (232) |
| ibuprofen | Oral | 755 | 465.9 | (224) | 463 | (91) | 460 | (74) |  | R05FA02 | (278) | N02BE01 | (211) | M03BB03 | (208) |
| ketorolac | Oral | 76 | 724.2 | (7) | 274.0 | (6) | 729.1 | (6) |  | A02AX-- | (24) | M03BB03 | (18) | A02AD03 | (14) |
|  | Parenteral | 364 | 401.9 | (62) | 733.00 | (62) | 715.90 | (29) |  | B05XA03 | (245) | N02BE01 | (140) | C01DA02 | (73) |
| meloxicam | Oral | 327 | 715.90 | (68) | 715.36 | (50) | 401.9 | (40) |  | N02BE01 | (90) | A02AA02 | (59) | A06AB06 | (59) |
| ketoprofen | Oral | 70 | 724.5 | (24) | 724.2 | (12) | 465.9 | (11) |  | A02AG-- | (31) | A02AA02 | (17) | A02BA01 | (17) |
|  | Parenteral | 125 | 401.9 | (62) | 733.00 | (62) | 715.90 | (29) |  | M01AB05 | (46) | A02BA01 | (40) | B05BA03 | (40) |
| piroxicam | Oral | 259 | 719.40 | (49) | 724.2 | (48) | 727.00 | (45) |  | A02AG-- | (137) | M01AB05 | (114) | M03BB03 | (103) |
| sulindac | Oral | 242 | 716.90 | (34) | 719.40 | (27) | 401.9 | (26) |  | M03BB03 | (83) | A02AG-- | (76) | N02BE01 | (72) |
| naproxen | Oral | 231 | 724.2 | (42) | 274.0 | (30) | 729.1 | (25) |  | M03BB03 | (116) | A02AG-- | (76) | M04AC01 | (69) |
| acemetacin | Oral | 349 | 715.90 | (34) | 401.9 | (27) | 274.0 | (26) |  | N05BX01 | (83) | A02AD03 | (76) | M03BB03 | (72) |
| indomethacin | Oral | 184 | 274.0 | (30) | 274.9 | (29) | 719.40 | (26) |  | M04AC01 | (152) | A02BA01 | (85) | N02BE01 | (66) |
| flurbiprofen | Oral | 129 | 724.2 | (23) | 274.0 | (14) | 681.9 | (13) |  | N05BX01 | (51) | A02AX-- | (37) | A02AG-- | (34) |
| tiaprofenic acid | Oral | 92 | 715.90 | (14) | 924.3 | (13) | 721.42 | (8) |  | N05BX01 | (44) | A02AD03 | (43) | A02AX-- | (20) |

(n) number of patients

Table S3: Most frequent diagnosis (ICD9-code) and most frequent drug (ATC code) concomitant prescriptions of the NSAIDs studied

| ICD9-CM code | Disease |  | ATC code | Ingredient name |
| --- | --- | --- | --- | --- |
| 460 | Acute nasopharyngitis [common cold] |  | A02AA02 | magnesium oxide |
| 463 | Acute tonsillitis |  | A02AD03 | aluminum dihydroxyallantoinate |
| 274.0 | Gouty arthropathy |  | A02AG-- | butylscopolamine bromide |
| 274.9 | Gout, unspecified |  | A02AX-- | sulcain (ethyl-p-piperidylacetylaminobenzoate) |
| 401.9 | Essential hypertension, unspecified |  | A02BA01 | cimetidine |
| 465.9 | Acute upper respiretory infections of unspecified site |  | A06AB06 | sennoside a+b (calcium) |
| 681.9 | Cellulitis and abscess of unspecified digit |  | A07EC01 | sulfasalazine |
| 714.0 | Rheumatoid arthritis |  | B05XA03 | sodium chloride |
| 724.2 | Lumbago |  | C01DA02 | nitroglycerin |
| 724.5 | Backache, unspecified |  | J01CA04 | amoxicillin |
| 729.1 | Myalgia and myositis, unspecified |  | M01AB05 | diclofenac sodium |
| 784.0 | Headache |  | M01AG01 | mefenamic acid |
| 924.3 | Contusion of toe |  | M03BB03 | chlorzoxazone |
| 715.36 | Osteoarthrosis, localized, not specified whether primary or secondary, lower leg |  | M04AC01 | colchicine |
| 715.90 | Osteoarthrosis, unspecified whether generalized or localized, unspecified site |  | M05BA04 | alendronate |
| 716.90 | Arthropathy, unspecified, unspecified site |  | N02BE01 | acetaminophen |
| 719.40 | Pain in joint, unspecified site |  | N05BX01 | mephenoxalone |
| 721.42 | Lumbar spondylosis with myelopathy |  | R05FA02 | dextromethorphan hbr |
| 727.00 | Synovitis and tenosynovitis, unspecified |  |  |  |
| 733.00 | Osteoporosis, unspecified |  |  |  |

Table S4: Number of patients received at least one prescription of NSAIDs studied in outpatient-clinic visit and total number of DDDs reimbursed by National Health Insurance Taiwan in 2006

|  | ATC Code | |  | | Patient number | % | Cumulative% | DDDs | Rank in DDDs |
| --- | --- | --- | --- | --- | --- | --- | --- | --- | --- |
| Oral DSAIDs | | | | |  |  |  |  |  |
| 1 | M01AB05 | | diclofenac sodium | | 7,273,719 | 29.3% | 29.3% | 100,393,915.12 | 1 |
| 2 | M01AG01 | | mefenamic acid | | 5,394,285 | 21.8% | 51.1% | 56,244,359.19 | 2 |
| 3 | M01AE01 | | ibuprofen | | 5,106,547 | 20.6% | 71.7% | 37,314,398.65 | 3 |
| 4 | M01AE02 | | naproxen | | 1,094,933 | 4.4% | 76.1% | 15,618,302.95 | 5 |
| 5 | M01AB11 | | acemetacin | | 946,596 | 3.8% | 79.9% | 13,118,383.97 | 7 |
| 6 | M01AC01 | | piroxicam | | 669,310 | 2.7% | 82.6% | 11,028,818.00 | 8 |
| 7 | M01AE09 | | flurbiprofen | | 518,621 | 2.1% | 84.7% | 5,871,701.62 | 11 |
| 8 | M01AB02 | | sulindac | | 499,048 | 2.0% | 86.7% | 8,102,476.31 | 10 |
| 9 | M01AB01 | | indomethacin | | 460,445 | 1.9% | 88.6% | 3,540,817.40 | 14 |
| 10 | M01AC06 | | meloxicam | | 405,038 | 1.6% | 90.2% | 15,618,006.30 | 6 |
| 11 | M01AE11 | | tiaprofenic acid | | 364,915 | 1.5% | 91.7% | 4,625,696.60 | 13 |
| 12 | M01AB15 | | ketorolac tromethamine | | 309,389 | 1.3% | 92.9% | 2,106,359.18 | 18 |
| 13 | M01AE03 | | ketoprofen | | 273,926 | 1.1% | 94.0% | 2,549,633.81 | 16 |
| 14 | M01AH01 | | celecoxib | | 254,840 | 1.0% | 95.1% | 19,722,785.92 | 4 |
| Total number of patients | | | | | 13,699,038 |  |  |  |  |
|  | | | | |  |  |  |  |  |
| Parenteral NSAIDs | | | | |  |  |  |  |  |
| 1 | | M01AB15 | | ketorolac tromethamine | 766,948 | 1,023,706 | | | 1 |
| 2 | | M01AE03 | | ketoprofen | 242,010 | 177,237 | | | 2 |
| 3 | | M01AB05 | | diclofenac sodium | 185,651 | 210,895 | | | 3 |
| Total number of patients | | | | | 1,127,886 |  | | |  |

Table S5: STROBE checklist

| Check |  | Item No | Recommendation |
| --- | --- | --- | --- |
| V | **Title and abstract** | 1 | (*a*) Indicate the study’s design with a commonly used term in the title or the abstract |
| V |  |  | (*b*) Provide in the abstract an informative and balanced summary of what was done and what was found |
|  | Introduction |  |  |
| V | Background/rationale | 2 | Explain the scientific background and rationale for the investigation being reported |
| V | Objectives | 3 | State specific objectives, including any prespecified hypotheses |
|  | Methods |  |  |
| V | Study design | 4 | Present key elements of study design early in the paper |
| V | Setting | 5 | Describe the setting, locations, and relevant dates, including periods of recruitment, exposure, follow-up, and data collection |
| V | Participants | 6 | *Case-control study*—Give the eligibility criteria, and the sources and methods of case ascertainment and control selection. Give the rationale for the choice of cases and controls |
| V |  |  | *Case-control study*—For matched studies, give matching criteria and the number of controls per case |
| V | Variables | 7 | Clearly define all outcomes, exposures, predictors, potential confounders, and effect modifiers. Give diagnostic criteria, if applicable |
| V | Data sources/ measurement | 8* | For each variable of interest, give sources of data and details of methods of assessment (measurement). Describe comparability of assessment methods if there is more than one group |
| V | Bias | 9 | Describe any efforts to address potential sources of bias |
| V | Study size | 10 | Explain how the study size was arrived at |
| V | Quantitative variables | 11 | Explain how quantitative variables were handled in the analyses. If applicable, describe which groupings were chosen and why |
| V | Statistical methods | 12 | (*a*) Describe all statistical methods, including those used to control for confounding |
| V |  |  | (*b*) Describe any methods used to examine subgroups and interactions |
| V |  |  | (*c*) Explain how missing data were addressed |
| V |  |  | *Case-control study*—If applicable, explain how matching of cases and controls was addressed |
| V |  |  | (*e*) Describe any sensitivity analyses |

Continued on next page

|  | Results |  |  |
| --- | --- | --- | --- |
| V | Participants | 13* | (a) Report numbers of individuals at each stage of study—eg numbers potentially eligible, examined for eligibility, confirmed eligible, included in the study, completing follow-up, and analysed |
| V |  |  | (b) Give reasons for non-participation at each stage |
| V |  |  | (c) Consider use of a flow diagram |
| V | Descriptive data | 14* | (a) Give characteristics of study participants (eg demographic, clinical, social) and information on exposures and potential confounders |
| V |  |  | (b) Indicate number of participants with missing data for each variable of interest |
| V | Outcome data | 15* | *Case-control study—*Report numbers in each exposure category, or summary measures of exposure |
| V | Main results | 16 | (*a*) Give unadjusted estimates and, if applicable, confounder-adjusted estimates and their precision (eg, 95% confidence interval). Make clear which confounders were adjusted for and why they were included |
| NA |  |  | (*b*) Report category boundaries when continuous variables were categorized |
| NA |  |  | (*c*) If relevant, consider translating estimates of relative risk into absolute risk for a meaningful time period |
| V | Other analyses | 17 | Report other analyses done—eg analyses of subgroups and interactions, and sensitivity analyses |
|  | Discussion |  |  |
| V | Key results | 18 | Summarise key results with reference to study objectives |
| V | Limitations | 19 | Discuss limitations of the study, taking into account sources of potential bias or imprecision. Discuss both direction and magnitude of any potential bias |
| V | Interpretation | 20 | Give a cautious overall interpretation of results considering objectives, limitations, multiplicity of analyses, results from similar studies, and other relevant evidence |
| V | Generalisability | 21 | Discuss the generalisability (external validity) of the study results |
|  | Other information |  |  |
| V | Funding | 22 | Give the source of funding and the role of the funders for the present study and, if applicable, for the original study on which the present article is based |
